# Grandparent–Grandchild Reminiscence Program for Asian American Families: Intergenerational and Cultural Reflection from Dyad Participants

**DOI:** 10.3390/bs16071248

**Published:** 2026-07-22

**Authors:** Ling Xu, Soeun Jang, Qinzheng Xu, Jaclyn E. Kirsch, Darren Yang

**Affiliations:** 1 School of Social Work, University of Texas at Arlington, 501 W Mitchell St, Arlington, TX 76010, USA; sxj7706@mavs.uta.edu (S.J.); jaclyn.kirsch@uta.edu (J.E.K.); 2 Industrial Economics, School of Business, The East China University of Political Science and Law, 555 Longyuan Road, Songjiang University Town, Shanghai 201620, China; wingerumu@hotmail.com; 3 Grapevine High School, 3223 Mustang Dr, Grapevine, TX 76051, USA; darrenbyang.30@gmail.com

**Keywords:** reminiscence therapy, grandparent–grandchild, psychological well-being, intergenerational relationships, narrative identity, Asian American families, cultural reflection

## Abstract

Background: Intergenerational solidarity between grandparents and grandchildren is a longstanding tradition that strengthens family bonds and supports the psychological wellbeing of older adults, particularly within Asian families. Drawing on theories of lifespan development, social connectedness, and narrative identity, an intergenerational reminiscence program was developed to promote emotional well-being and deepen generational connections among Asian American families. Methods: This study qualitatively examined the reflections of 12 grandparent–grandchild dyads who completed the program. Grandparents participated in six weekly, one-hour guided life review and reminiscence sessions, conducted either in person or online and facilitated by grandchildren. Following the intervention, individual interviews were conducted with the dyads. Interview data were analyzed using Braun and Clarke’s six-step thematic analysis approach. Results: Three themes emerged describing participants’ experiences with intergenerational storytelling and learning. The first, engagement with family history and stories, captured participants’ enjoyment of sharing and discovering family narratives, uncovering previously unknown histories, and building curiosity about their ancestry. The second theme, transmission of wisdom and life lessons, reflected the intergenerational sharing of career guidance, practical life advice, perspectives on resilience, and strengthened appreciation for personal circumstances. The third theme, intergenerational and cultural understanding, highlighted increased awareness of generational differences, respect for cultural and personal viewpoints, and strengthened emotional bonds. Conclusions: The findings align with cultural transmission theory, intergenerational solidarity theory, and psychological theories of reminiscence and identity formation. Reflections from both grandparents and grandchildren suggest that intergenerational reminiscence effectively fosters closer relationships across generations by revisiting family history and cultural roots. Lessons learned and recommendations for future culturally responsive interventions with Asian American families are discussed.

## 1. Introduction

### 1.1. Older Asian Americans

Asian Americans represent one of the fastest-growing ethnic groups in the United States, with the proportion of older adults projected to rise from 4.6% in 2019 to 8% by 2060 (Administration for Community Living, 2020). Despite this demographic growth, older Asian Americans experience distinct emotional and psychological challenges that are often overlooked. Asian cultural values emphasizing family cohesion, self-reliance, and endurance may contribute to anxiety, reduced self-esteem, and emotional distress (Guo et al., 2015, 2016; Sastry & Ross, 1998). Mental health struggles may be perceived as signs of personal weakness, which can reinforce internalized stigma and deter individuals from seeking help (Yang & Woo, 2025).

Older Asian Americans also underutilize formal mental health services compared to their White counterparts. Barriers such as limited English proficiency, cultural stigma surrounding mental health treatment, and preference for traditional healing practices contribute to persistent unmet mental health needs (Kramer et al., 2002). Yet these challenges are rarely explored through an intergenerational lens, despite the centrality of family relationships in Asian cultures. Enhancing emotional well-being and intergenerational closeness is particularly relevant to mental health among Asian American older adults, as emotional isolation, weakened family connection, and limited opportunities for meaningful expression are closely linked to psychological distress and underutilization of mental health services in this population (Abe-Kim et al., 2007; Dong et al., 2012; G. Kim et al., 2011; Li & Dong, 2018).

### 1.2. Intergenerational Issues Within Asian Immigrant Families

Although family cohesion and interdependence are central values in many Asian cultures, these ideals do not always translate into emotional closeness or effective communication within Asian American immigrant families. In immigrant contexts, this apparent contradiction reflects a gap between strong cultural expectations of family closeness and the lived realities of intergenerational communication shaped by migration, acculturation, and language differences (L. Choi, 2022; Ho & Birman, 2010; S. Y. Kim et al., 2013; Tsai-Chae & Nagata, 2008). In Asian American families, grandparent–grandchild relationships are shaped by cultural, generational, and acculturation-related factors that can present both opportunities and tensions (Bhandari, 2019; Xu et al., 2018). Acculturation gaps, stemming from differences in cultural values, social expectations, or behavioral norms, frequently underlie misunderstandings. Grandparents who retain cultural traditions from their country of origin may emphasize collectivism, filial piety, and adherence to established customs, while U.S.-born grandchildren may lean toward more individualistic perspectives (Y. Choi et al., 2013; Foner & Dreby, 2011; Hwang, 2006; Xu et al., 2018. These contrasts can contribute to conflicting beliefs about roles, obligations, and appropriate behaviors within the family (L. Choi, 2022; Sung, 2001; Zhang & Slaughter-Defoe, 2009).

Perceived emotional distance may further complicate intergenerational bonds. While grandparents may express affection through culturally meaningful actions, grandchildren may interpret these as a lack of emotional openness (L. Choi, 2022; Guo et al., 2015; Louie, 2006). Language barriers also remain a prominent obstacle: many grandparents have limited English proficiency, while grandchildren may have lower proficiency in the family’s heritage language. These linguistic gaps restrict nuanced communication, hinder the transmission of cultural knowledge, and can widen intergenerational distance (Louie, 2006; Tse, 2001). It is also important to note that these intergenerational dynamics are not uniform across Asian American families and may vary by national origin, migration history, and cultural context, with patterns observed in Chinese American families differing in important ways from those in Indian, Vietnamese, or other Asian American groups. Collectively, these dynamics highlight the need for culturally responsive programs that promote communication, understanding, and emotional closeness across generations.

### 1.3. Intergenerational Reminiscences Approach

Reminiscence involves structured reflections on past events, experiences, and personal life stories, often supported by prompts such as photographs or music (Pinquart & Forstmeier, 2012). As an intervention, reminiscence has been shown to reduce depressive symptoms (Chen et al., 2012; Karimi et al., 2010; Song et al., 2014) and enhance emotional and social wellbeing among older adults (Meléndez et al., 2015; Pinquart & Forstmeier, 2012; Pishvaei et al., 2015; Woods et al., 2018). When paired with intergenerational engagement, reminiscence may confer additional benefits by strengthening relationships and providing structured opportunities for cultural and life-history transmission (Chung, 2009; Manohar et al., 2023). Evidence suggests that intergenerational reminiscence reduces social isolation for both generations, improves attitudes across generations, and builds empathy and mutual understanding (Gardiner et al., 2018; Webster & McCall, 1999).

However, most existing intergenerational reminiscence programs pair older adults with student volunteers rather than family members (Gerritzen et al., 2020). Few interventions directly engage grandparents and grandchildren, despite the natural relational context and sustainability afforded by family-based models. Involving grandchildren as conversation partners eliminates reliance on trained professionals, reduces program costs, and provides meaningful opportunities for family bonding, particularly beneficial in community or resource-limited settings.

### 1.4. The Present Study

To address these gaps, a six-session life-review program grounded in reminiscence therapy was developed for Asian American older adults, guided by theories of lifespan development, social connectedness, and narrative identity. Grandparents participated in weekly reminiscence conversations led by one of their grandchildren, with the goal of enhancing emotional wellbeing and intergenerational closeness. The overall project employed a mixed-methods study design. Quantitative data (pre- and post- intervention surveys) and qualitative data (weekly reflections and postintervention interviews) were collected to assess changes in wellbeing and relational dynamics. Details of the intervention design are described in the published protocol (Xu et al., 2023).

The aim of the present paper is to explore how Asian American older adults and their grandchildren experience an intergenerational reminiscence-based program, with particular attention to relational dynamics and cultural continuity, as reflected in the experiences of participating dyads. To address this aim, the current analysis exclusively focuses on qualitative findings from postintervention interviews, which were designed to elicit participants’ holistic reflections on relational change, emotional well-being, and cultural meaning-making following completion of the program. Weekly reflections served primarily as process-oriented data to support intervention fidelity and participant engagement and are therefore not the focus of the current analysis.

## 2. Materials and Methods

### 2.1. Intervention Process

To ensure consistency and fidelity in program delivery, grandchildren received a structured training session and an intervention manual prior to beginning the life-review conversations. Training was delivered online in small groups of one to two grandchildren and lasted approximately one hour. The intervention manual was adapted from the reminiscence frameworks developed by Watt (Watt, 1996) and Watt and Cappeliez (Watt & Cappeliez, 2000), which integrate elements of integrative reminiscence (focused on meaning-making, identity, and life continuity) and instrumental reminiscence (drawing on past experiences to address current challenges). The combined approach aimed to promote self-acceptance, conflict resolution, reconciliation, a sense of meaning and self-worth, and the ability to recall and apply past coping strategies.

The training introduced grandchildren to the six life-review themes and provided guidance on facilitating meaningful, respectful, and culturally sensitive conversations with their grandparents. The intervention consisted of six weekly sessions, each approximately one hour, with one structured theme per week: (1) Major turning points in life; (2) Family history; (3) Life and career accomplishments; (4) History of likes and dislikes; (5) Experiences of stress and coping strategies; (6) Reflections on meaning and purpose in life.

These themes were designed to promote self-reflection, elicit storytelling, and facilitate intergenerational bonding. The ultimate goals were to enhance emotional well-being among older adults and strengthen relational closeness between grandparents and grandchildren. To maintain engagement and adherence, the research team conducted brief weekly check-in calls with each dyad. These calls, lasting approximately five minutes, provided opportunities to address questions, offer technical support, and ensure smooth implementation of sessions. Pre- and post- intervention surveys were administered to all participants, and interviews were conducted with a convenience sample of dyads following program completion.

### 2.2. Participants

Participants were recruited from the Dallas–Fort Worth (DFW) metropolitan area on a rolling basis. Eligibility criteria required older adults to self-identify as Asian American and be of Chinese, Korean, Vietnamese, or Indian descent. Older adult participants were aged 65 years or older and either self-reported cognitive challenges or were identified by caregivers as experiencing such difficulties. Each older adult was required to have at least one grandchild aged 18 years or older who was willing and able to participate. The severity of cognitive impairment was not formally assessed. However, all older adult participants were capable of providing informed consent and communicating effectively during interviews, indicating that participants were generally functioning at a normal level of cognitive function, with no more than mild cognitive impairment or subjective cognitive concerns. To facilitate meaningful communication during the reminiscence sessions, both members of each dyad needed to share proficiency in at least one common language (English or a heritage language). This requirement ensured conversational fluidity, cultural relevance, and the ability to engage authentically in storytelling.

A convenience sampling strategy was used for the postintervention interviews. The final sample comprised 12 dyads who completed the intervention and consented to and were available for the interviews, representing approximately half of the 26 dyads enrolled in the study. The interview sample included one Vietnamese family, one Vietnamese and Chinese family, and one Indian family, while the remaining nine dyads were Chinese American families (see Table 1 and Table 2).

### 2.3. Data Collection and Interview Guideline

The study was approved by the Institutional Review Board of the authors’ institution (IRB# 20200210). The intervention occurred between January 2021 and December 2022 and the postintervention interviews were conducted within one month after the intervention. Because this paper reports qualitative findings from postintervention interviews, only the qualitative interview data collection procedures are described below.

At the conclusion of the six-session program, semi-structured interviews were conducted via Microsoft Teams with dyads (separately with grandparent and grandchild) who completed the 6-week intervention. Postintervention interviews were conducted by three trained bilingual research assistants (RAs) hired for the project, with each RA facilitating interviews with participants from a specific ethnic group (Chinese, Vietnamese, or Indian). Interviews with grandparent and grandchild were conducted separately, and in participants’ preferred language (English or a heritage language). Interviews explored perceived changes in emotional well-being, shifts in intergenerational relationships, and reflections on cultural identity and family heritage. Interviews lasted 45–60 min, providing rich and detailed insights into dyadic experiences. This structured yet flexible format ensured consistency across interviews while allowing participants to share personal experiences in depth.

### 2.4. Data Analysis

Interview transcripts were analyzed using Braun and Clarke’s (Braun & Clarke, 2006) six-step thematic analysis, which provides a systematic and rigorous framework for identifying patterns within qualitative data. Content analysis of the interview transcripts was conducted using Atlas.ti version 23 (ATLAS.ti Scientific Software Development GmbH, 2023, Berlin, Germany).

(1) Familiarization: The research team read transcripts multiple times and took preliminary notes to gain an overall understanding of the dataset. The familiarization process revealed recurring discussions of family history, advice-giving, cultural differences, and relational change.
(2) Generating initial codes: Two researchers (authors of this manuscript) independently conducted line-by-line coding of data segments relevant to the research aims using an inductive approach. Initial coding captured participants’ reflections related to storytelling, emotional reactions, transmission of values, and intergenerational interactions.
(3) Searching for themes: Codes were organized by the same coders in above step into broader conceptual categories, such as learning about family history, sharing life lessons, and navigating generational and cultural differences, and potential themes reflecting shared patterns across dyads were identified in team meetings.
(4) Reviewing themes: Emerging themes were examined in relation to both coded excerpts and the entire dataset to ensure internal coherence and alignment with the data.
(5) Defining and naming themes: Through iterative review and team discussion themes were refined, clarified, and defined to capture distinct and substantive aspects of participants’ experiences.
(6) Producing the report: Final themes were synthesized into an analytic narrative supported by illustrative participant quotations.

In this analytic process, no a priori codebook based on a theoretical framework was developed prior to reviewing the interview transcripts. Reflexivity was maintained through ongoing team discussions that considered how researchers’ disciplinary backgrounds and prior experience with Asian American families might influence analytic decisions. Strategies to enhance trustworthiness included investigator triangulation, iterative review of codes and themes, and maintaining an audit trail of analytic decisions. Data saturation was reached when no new themes emerged and thematic patterns were consistent across dyads.

## 3. Results

The analysis revealed three overarching themes that captured participants’ experiences of intergenerational storytelling and learning. The first theme, engagement with family history and stories, reflected participants’ enjoyment in hearing about grandparents’ childhoods, family relationships, and cultural experiences, as well as their curiosity about previously unknown aspects of their family history. The second theme, transmission of wisdom and life lessons, highlighted the guidance grandparents shared, including career advice, resilience strategies, positive attitudes toward life, and a deeper appreciation of personal circumstances. The third theme, intergenerational and cultural understanding, emphasized participants’ recognition of generational differences, respect for cultural and personal perspectives, and strengthened family bonds as a meaningful source of support. Together, these themes illustrate how storytelling fosters personal growth, deepens intergenerational connection, and enhances cultural awareness and appreciation within families.

Although all three themes were evident across both grandparents and grandchildren, grandchildren’s perspectives are more frequently illustrated in the presented quotations. This reflects that grandchildren were generally more willing and enthusiastic to share their experiences during the postintervention interviews, whereas grandparents had additional opportunities to provide feedback through pre- and postintervention surveys. As a result, grandchildren’s interview narratives more often emphasized reflections on learning, relational change, and personal impact. Additionally, the results primarily reflect the experiences of Chinese American grandparent–grandchild dyads, with more limited representation from Vietnamese and Indian families, and should be interpreted in light of this sample composition. The qualitative findings primarily reflect participants’ perceived benefits and reflections following the intervention; fewer accounts described challenges, tensions, or negative experiences during the conversations.

### 3.1. Theme 1: Engagement with Family History and Stories

#### 3.1.1. Enjoyment of Family Narratives

Participants who were grandchildren valued hearing stories about family life, childhood experiences, and sibling relationships from their grandparents.

The ‘family life’ one, that’s week 2. The child, her childhood. That I enjoy most. Ever since I was young, I actually enjoyed listening to her tell me stories and some of the things she repeated, some of the stories were new. … I’m an only child, so I think it’s just the idea of having siblings and having, I guess, seeing how she deals with them. … I just find it interesting, and I enjoy listening to these things. And I enjoy kind of seeing the life she grew up in and kind of comparing it to what I have now and how much my life is different environment wise, culture wise. And to see I guess also to see the impact of how she treats situations around her like uh some of the cultural impacts.

(Family #1, Granddaughter, age 18)

#### 3.1.2. Discovery of Unexpected or Unknown Family History

Grandchildren gained new insights about grandparents’ upbringing, challenges, and perspectives previously unknown.

I think it had given me more insight regarding the upbringing of my mother and the various family relationships that my grandparent had at that time. I felt the same emotions that she had at the time, frustration and anger for those that had taken advantage of her. Although I thought I knew a lot about my mom’s childhood, my grandma had told me her perspective and what she could’ve done instead.

(Family #3, Granddaughter, age 20)

Additionally, grandparents reported that they were given the opportunity to provide more details about their life to their grandchildren.

Children can ask a lot of questions, and I answered many of them. She (granddaughter) said, there were many things that I didn’t talk about at home before. We mostly communicated through written words, but this time I shared a bit more, and went a bit deeper.

(Family #3, Grandmother, age 77)

#### 3.1.3. Curiosity and Anticipation to Learn More

Grandchildren expressed a desire to learn more about cultural influences, family relationships, and life lessons.

I want to know more about her idea on culture, how much she sees that culture impacts her actions when she was still in China, when she came in, immigrated over here and later in life when she’s interacting with, with me or with other people. I feel like it’d be like a very interesting discussion to have with her.

(Family #6, Grandson, age 21)

I would love to hear more about my grandma’s siblings in China; how she got to know my grandpa and got married eventually, like lessons on successful marital relationship kind of.

(Family #9, Granddaughter, age 19)

### 3.2. Theme 2: Transmission of Wisdom and Life Lessons

#### 3.2.1. Career and Practical Guidance

Grandparents shared insights about work ethic, prioritization, honesty, and leadership.

In my career, I’ve learned how to lead people and how to get things done. I shared a lot with her (granddaughter) about this. Through it, I realized that being honest is the most important quality, and that one’s words and actions should be in harmony. This way, we feel at ease, and so do others. If you say one thing today and do another tomorrow, or switch from a Chinese style one day to an American style the next, it creates confusion and ends up in a mess. So, I believe the most important thing is to be consistent inside and out, and to treat others with honesty. I really want her to learn this from my experience.

(Family #3, Grandmother, age 77)

I told [my grandson] that for the things I want to do, I feel it’s necessary to prioritize them. Complete the most important tasks first, then move on to the next one, tackling each task one by one. And I shouldn’t be influenced by external distractions.

(Family #5, Grandfather, age 83)

#### 3.2.2. Attitude Towards Life and Personal Growth

Lessons on positivity, self-confidence, and resilience were emphasized.

Well, she kind of taught me that like, do you like more positive about things? Cause if you’re like not positive, then you’re not really gonna have fun. If you act positive about something you might find is actually fun.

(Family #10, Granddaughter, age 18)

Her main lesson was to have self-confidence, to be able to believe in yourself that you will get through it and, if you need help, you should ask for help like there are people who are willing to be out there to help you if you need it, but mainly it’s to believe in yourself and to believe that.

(Family #4, Granddaughter, age 20)

#### 3.2.3. Resilience and Coping

Participants highlighted learning from grandparents’ ability to overcome challenges and face adversity.

Because some of her situations were, I guess traumatic. But for her, she was like, oh, I’ve passed through it. No problem. So I think she wants me to know, I can get through everything. I’ve come to realize that a lot of times schools don’t teach you how to face real world situations. And my grandmother has taught me that no matter what the real-world situation is, you have to brace yourself and adjust.

(Family #11, Granddaughter, age 21)

I think I felt, determined a bit, because she’s like, like a role model, I would say, because her life was a lot tougher than mine. And then she had to work through a lot of situations, which I have the luxury of not doing. But I felt determined to work hard and just to work hard and do better in my life.

(Family #12, Grandson, age 18)

#### 3.2.4. Appreciation and Perspective

Participants developed appreciation for their current circumstances and family support which they learned from their grandparents.

My grandma was at the time [compared to] everyone was poor. No one had a room. They had just one room with one wooden bed, and everyone would all sleep on that bed. So it’s an eye opener to see how much of a better life I have in comparison to her. And how much I don’t have to work for versus how much I do have to work for.

(Family #11, Granddaughter, age 21)

### 3.3. Theme 3: Intergenerational and Cultural Understanding

#### 3.3.1. Difference Between Generations

Generational differences in priorities, approaches to life, and learning were noted.

When I suggest that they should put more effort into studying Chinese, they seem to think it’s not that important or a priority right now. I still feel that it’s quite necessary, so I give some examples to support my point. However, they don’t seem to take it very seriously.

(Family #5, Grandfather, age 83)

My grandpa had a focus on spending like 100% of life on working hard and fighting for success. But as in my generation it’s like yeah maybe I’ll spend 70% of my time working hard and fighting for success. The other 30% more just enjoying life.

(Family #5, Grandson, age 26)

I would have been more decisive in a lot of situations, but for her she kind of thought the bigger picture and that’s kind of how she lives her life, and oftentimes she neglects her own feelings or her own identity, I guess. And I think that’s where my ideas differ like, I don’t think I can satisfy everyone else and hurt myself in the long run.

(Family #6, Granddaughter, age 21)

#### 3.3.2. Cultural Respect and Mutual Understanding

Participants reflected on navigating cultural differences and respecting each other’s perspectives.

When he changes jobs or makes other decisions, I hope he approaches it cautiously, but he feels it’s no big deal and isn’t worried, he has his own perspective. At times, we have some differences in opinion about this. However, overall, I respect their choices, whether it’s my daughter, son-in-law, or grandson. I do share my own views or past experiences, though they might not necessarily apply to them.

(Family #10, Grandpa, age 83)

#### 3.3.3. Strengthened Family/Kinship Bonds

Participants recognized family as a source of support, guidance, and shared identity.

I learned that like whenever you’re facing like issues or problems in your life that you always have, like your family to rely on. Before I was probably thinking like if you really needed help, you probably could go to like a therapist or something. But I don’t know why I just never thought of family as like a support system if you were having issues.

(Family #12, Grandson, age 18)

## 4. Discussion

The present study explored the experiences of grandparent–grandchild dyads engaging in intergenerational reminiscence conversations, highlighting how family narratives foster personal growth, cultural understanding, and intergenerational bonding. Three major themes emerged from the qualitative analysis: engagement with family history, transmission of wisdom and life lessons, and intergenerational and cultural understanding. These findings underscore the multifaceted role of grandparents in shaping not only the individual development of their grandchildren but also the continuity of family culture and values. Given that the majority of participants were Chinese American families, the findings should be interpreted as most directly reflecting the experiences of this group, while offering insights that may be relevant, but not uniformly applicable, to other Asian American families.

These findings are consistent with cultural transmission theory that focuses on how cultural values, beliefs, and behaviors are passed down from one generation to the next. Based on this theory, in immigrant families, grandparents play a significant role in transmitting cultural heritage, but this transmission is often challenged by the broader societal context in which grandchildren are socialized (Cavalli-Sforza & Feldman, 1981). The theory highlights factors such as language barriers, the influence of the host culture, and generational attitudes toward cultural identity can affect how much of the heritage culture is preserved or modified. On the other hand, these findings also confirm the intergenerational solidarity theory that looks at the bonds between family members across generations, emphasizing dimensions like affectual solidarity (emotional closeness) consensual solidarity (shared values), and functional solidarity (exchange of support). In Asian immigrant families, cultural values around family obligations and expectations of respect and support for older adults can shape intergenerational solidarity. However, the level of shared values or emotional closeness may vary depending on each generation’s cultural orientation, leading to varying degrees of intergenerational solidarity or conflict (Bengtson & Roberts, 1991).

### 4.1. Grandparents as Key Cultural Transmitters

One of the most salient findings was the role of grandparents as primary transmitters of cultural knowledge and family heritage. In many Asian immigrant families, grandparents serve as key cultural transmitters, bringing with them knowledge of heritage traditions, customs, and native language that might otherwise be diluted or lost over generations. Grandparents often feel a strong responsibility to pass down cultural values, morals, and practices, such as respect for elders, perseverance, and communal values, which are integral to their heritage. This transmission can give grandchildren a deeper sense of family history, grounding them in values that persist despite acculturation pressures. In this study, grandchild participants consistently described the enjoyment and curiosity they experienced while listening to stories about their grandparents’ childhoods, family relationships, and life experiences. These narratives provided a window into the cultural and historical context of previous generations, allowing grandchildren to situate their own lives within a broader familial and societal framework. For example, one participant reflected on how stories about siblings and childhood routines revealed cultural norms and family dynamics previously unknown to them, highlighting the richness of lived experience that grandparents carry. These findings are consistent with prior research showing that grandparents in immigrant families often function as custodians of cultural memory, family history, and practices that shape familial identity to younger generations despite acculturation pressures (Cavalli-Sforza & Feldman, 1981; L. Choi, 2022; Fivush, 2019; Guo et al., 2015). Extending this literature, the family-based reminiscence context appeared to deepen this transmission by embedding cultural learning within emotionally meaningful dyadic exchanges. 

### 4.2. Transference of Values and Traditions

Beyond cultural knowledge, the conversations revealed the transference of values, moral lessons, and life strategies from grandparents to grandchildren. Participants frequently cited guidance on career ethics, prioritization, honesty, and integrity as critical lessons imparted during these intergenerational dialogues. Additionally, grandparents modeled attitudes towards life, emphasizing positivity, self-confidence, and the importance of resilience in the face of adversity. These teachings extended beyond practical advice, encompassing broader ethical and relational frameworks that participants could incorporate into their own decision-making processes. These findings are consistent with prior research indicating that intergenerational storytelling functions as a key mechanism of socialization, through which older family members transmit moral reasoning, work ethic, and culturally grounded values to younger generations (Bengtson & Roberts, 1991; Cavalli-Sforza & Feldman, 1981; Silverstein et al., 2025). In immigrant families, such value transmission is particularly salient, as grandparents often serve as anchors of continuity amid acculturation-related shifts in norms and expectations (Foner & Dreby, 2011; Guo et al., 2015).

### 4.3. Strengthening Family Bonds Through Cultural Continuity

When grandparents and grandchildren engage in cultural practices together, such as learning family stories, the family bond is strengthened. This shared cultural experience enhances a collective family identity, creating a sense of belonging across generations. The process of storytelling also played a crucial role in strengthening intergenerational bonds. Learning family stories fostered empathy, understanding, and mutual respect between generations. Participants noted that hearing personal accounts of grandparents’ challenges and triumphs deepened their appreciation for family members and encouraged a more nuanced understanding of their grandparents’ perspectives. Grandchildren also reported being greatly impacted by the experience as they learned unknown and unexpected family history, which helped strengthen their perspective on family and overall cultural values. Emotional reactions ranged from experiencing empathy to admiration, overall concluding the result of a deeper connection and emotional intimacy. Such experiences facilitated emotional closeness and provided opportunities for grandchildren to reflect on their own family relationships, bridging generational gaps. Prior studies similarly suggest that shared family narratives and intergenerational storytelling strengthen emotional closeness and family cohesion by fostering empathy, mutual understanding, and a shared sense of identity across generations (Bengtson & Roberts, 1991; Fivush, 2019; Foner & Dreby, 2011). Compared with intergenerational programs involving non-family members (Chung, 2009; Gerritzen et al., 2020), the dyadic family context may have amplified relational meaning and sustainability of these bonds. While grandchildren frequently reflect on learning from grandparents’ guidance and on perceived shifts in relational closeness, these findings represent subjective postintervention reflections rather than demonstrated long-term behavioral or relational change.

### 4.4. Building Intergenerational Resilience

A particularly notable theme was the cultivation of resilience across generations. Findings often show that both generations benefit emotionally from this resonance, as it promotes resilience and mental well-being. For grandchildren, knowing their family’s cultural legacy and seeing the resilience in their grandparents’ stories provides a sense of purpose and strength. Grandparents, in turn, feel valued and purposeful in sharing their experiences, reducing feelings of isolation or displacement. Grandchildren observed and internalized their grandparents’ strategies for coping with adversity, including perseverance, adaptability, and emotional regulation. By sharing experiences of overcoming hardship, grandparents modeled resilience in a manner that was concrete and relatable for younger family members. Participants described feeling inspired to face their own challenges with greater confidence and determination, indicating that these intergenerational exchanges had an impact on their personal growth. Resilience, in this context, is not only an individual trait but a relational and culturally embedded process, facilitated by the transmission of experiential knowledge and ethical guidance (Ungar, 2019). Consistent with resilience frameworks emphasizing relational and cultural resources, grandparents’ narratives of adversity provided grandchildren with culturally grounded models for coping and meaning making (Ungar, 2019). This finding also aligns with evidence that intergenerational exchanges can buffer stress and support psychological well-being across generations, particularly in families shaped by migration-related challenges (El-Khalil et al., 2025).

### 4.5. Practical Implications

The study’s results have practical implications for programs aimed at strengthening intergenerational relationships. Interventions that facilitate structured storytelling or dialogue between grandparents and grandchildren may enhance cultural continuity, relational closeness, and emotional support within families. For example, family-centered educational or social programs could encourage older adults to share narratives that convey both historical context and personal wisdom, thereby promoting intergenerational learning and resilience. Intergenerational trauma carries documented multigenerational consequences for family systems, affecting psychological well-being, relational patterns, and cultural identity across generations (El-Khalil et al., 2025). Emerging evidence suggests that strengthening intergenerational bonds may serve as a protective mechanism, with interventions targeting cross-generational connection associated with reduced transmission of trauma sequelae and improved well-being outcomes (Isobel et al., 2019). Furthermore, these findings underscore the value of considering cultural and familial context in designing interventions for both older adults and younger family members, as the mutual benefits extend to identity formation, coping strategies, and life satisfaction.

### 4.6. Limitations and Future Directions

While the study provides rich insights into intergenerational learning, several limitations should be noted. First, the sample size was limited, and participants were primarily drawn from families with some level of existing grandparent–grandchild engagement. Though the structured reminiscence format introduced intentional and guided opportunities for life review, emotional expression, and intergenerational reflection, the extent to which these benefits differ for families with lower baseline engagement warrants further investigation.

Second, the sample was predominantly composed of Chinese American families, which limits the transferability of findings to other Asian American groups with distinct cultural, linguistic, and migration histories. Although Asian American families share some broad cultural values, intergenerational experiences are not uniform and may vary by national origin, migration history, and cultural context. Additionally, the sample was geographically constrained to the DFW area, which may not represent the experiences of Asian families in other regions of the United States or internationally. Future research could explore a more diverse population across different Asian ethnicities and geographic locations to enhance generalizability.

Third, self-reported data may be subject to recall bias or social desirability effects. Participants may have emphasized positive aspects of the experience due to social desirability. This tendency may be further shaped by cultural norms in many Asian families that value harmony, respect for elders, and the avoidance of open conflict, which may discourage explicit discussion of tension, discomfort, or dissatisfaction during intergenerational interactions.

Fourth, because data were collected shortly after the intervention, the study cannot assess whether perceived changes in relationships or learning were sustained over time. Longitudinal designs could examine how intergenerational interactions influence development over time. Additionally, cognitive impairment severity was not formally measured, which limits our understanding of how varying levels of cognitive difficulty may have influenced participation or interpretation of the qualitative findings.

Lastly, although the study employed a dyadic design, grandparents and grandchildren were interviewed separately using different interview guides, which limited our ability to conduct direct within-dyad comparisons of how specific conversations were experienced by each member. Future studies could incorporate paired or joint interviews and aligned questioning to more fully examine convergences and divergences in dyadic perspectives and relational change.

## 5. Conclusions

Overall, the study reports that grandparents play a pivotal role in transmitting culture, values, and resilience to younger generations. Through the sharing of personal narratives and lived experiences, grandchildren gain insight into family history, develop life skills, and strengthen relational bonds. These findings underscore the importance of intergenerational dialogue as a mechanism for fostering cultural continuity, ethical development, and psychological resilience, highlighting the importance of grandparents as educators, role models, and cultural custodians within the family system. Although the findings highlight benefits for younger generations, intergenerational reminiscence also supported older adults’ emotional well-being by fostering meaningful expression and strengthened relational connection, both of which are closely linked to mental health in later life. Together, these findings underscore the bidirectional value of intergenerational reminiscence for both grandparents and grandchildren.

## Figures and Tables

**Table 1:** Demographic Information of Grandparents.

Family ID	Age	Education	Gender	Ethnicity	Marital Status	Years in the U.S.	Living Arrangement	Self-Rated Health
1	75	High school	F	Chinese	W	50	Alone	Very good
2	88	High school	F	Chinese	W	36	Alone	Fair
3	77	High school	F	Chinese	M	35	With spouse	Very good
4	86	Bachelor’s degree	M	Chinese	W	20	Alone	Fair
5	83	Bachelor’s degree	M	Chinese	M	10	With spouse	Good
6	86	Less than high school	F	Vietnamese	M	15	With children	Fair
7	82	High school	F	Indian	M	38	With spouse	Fair
8	72	Less than high school	F	Vietnamese	M	43	With spouse	Good
9	87	High school	F	Chinese	M	20	With spouse and children	Good
10	83	Bachelor’s degree	M	Chinese	W	19	Alone	Very good
11	78	Graduate or professional	F	Chinese	M	33	With spouse	Very good
12	72	High school	F	Chinese	M	4	With spouse	Very good

Note: under “Gender”: M = male, F = female. This is the same for Table 2 below; under “Marital status”: M = married; W = widowed.

**Table 2:** Demographic Information of Grandchildren.

Family ID	Age	Education	Gender	Ethnicity	U.S. Born	Geographic Distance with GP	Self-Rated Health
1	18	High school	F	Chinese/ Vietnamese	Yes	Another state within the USA	Excellent
2	26	Bachelor’s degree	F	Chinese	Yes	The same state	Good
3	20	Some college	F	Chinese	Yes	The same state	Good
4	20	Some college	F	Chinese	Yes	Another state within the USA	Very good
5	26	Bachelor’s degree	M	Chinese	No	Another state within the USA	Very good
6	21	Some college	F	Vietnamese	Yes	The same state	Good
7	25	Some college	M	Indian	Yes	Another state within the USA	Good
8	22	Bachelor’s degree	M	Vietnamese	Yes	The same state	Good
9	19	Some college	F	Chinese	Yes	The same state	Good
10	18	High school	F	Chinese	Yes	The same city	Excellent
11	21	Some college	F	Chinese	Yes	The same house	Good
12	18	High school	M	Chinese	Yes	Another state within the USA	Very good

## Data Availability

The data presented in this study are available on request from the corresponding author. The data are not publicly available due to its qualitative nature.
